# Virtual Reality Meditation Among Youth Experiencing Homelessness: Pilot Randomized Controlled Trial of Feasibility

**DOI:** 10.2196/18244

**Published:** 2020-09-24

**Authors:** Laura Johnson Chavez, Kelly Kelleher, Natasha Slesnick, Eugene Holowacz, Ellison Luthy, Laura Moore, Jodi Ford

**Affiliations:** 1 Nationwide Children's Hospital Columbus, OH United States; 2 Department of Human Sciences The Ohio State University Columbus, OH United States; 3 College of Nursing Columbus, OH United States

**Keywords:** virtual reality, meditation, homelessness, stress

## Abstract

**Background:**

Homelessness among youth is devastating, with high rates of substance use disorders and mental health comorbidity. Mindfulness-based interventions that include meditation and mindfulness skills training reduce stress and symptoms of anxiety or depression. However, engaging high-risk youth in interventions is challenging. Virtual reality is a more flexible platform for delivering meditation and may be appealing to youth.

**Objective:**

The study objectives were to evaluate the feasibility of delivering virtual reality meditation and of collecting outcome measures, including anxiety and physiologic stress.

**Methods:**

A sample of 30 youth experiencing homelessness was enrolled in the study. Youth were randomized to receive 10 minutes of one of three interventions: (1) virtual reality meditation, (2) audio meditation (through a web-based platform), or (3) virtual reality imagery of historical pictures and text. Subjects who consented to the study attended two research visits. The first visit collected survey measures of demographics, mental health, and substance use, and oriented subjects to the intervention platforms. The second visit (1-3 days later) delivered the intervention and collected pre and post outcome measures of anxiety and physiologic stress (salivary cortisol). Changes in anxiety and cortisol at the second visit were compared across groups using a linear regression model in which the primary analysis compared virtual reality meditation to audio meditation and secondary analyses compared virtual reality meditation to virtual reality imagery.

**Results:**

Anxiety scores decreased in all groups, with a larger reduction among the virtual reality meditation group (difference=10.8) compared to the web-based meditation or virtual reality images groups (difference=5.8 and 5.0, respectively). After controlling for baseline values, there were no significant group differences in changes in anxiety scores or cortisol levels. In comparing virtual reality meditation and audio meditation, the effect size for anxiety was moderate (Cohen *d*=0.58) while the effect size for cortisol was small (Cohen *d*=0.08).

**Conclusions:**

Preliminary results suggest that virtual reality meditation has a moderate benefit for anxiety but not physiologic stress. Future research is needed to confirm these results in a larger sample and to investigate whether the effects are sustained or increase with repeated use of virtual reality mediation. Virtual reality meditation appears feasible to deliver among homeless youth and merits further study.

**Trial Registration:**

ClinicalTrials.gov NCT04004520; https://clinicaltrials.gov/ct2/show/NCT04004520

## Introduction

Youth homelessness is common in the United States, with an estimated 3.5 million young adults experiencing homelessness in a given year [1]. Youth who experience homelessness have a high prevalence of mental health conditions [2]. Many homeless youth have experienced physical or sexual abuse prior to or after becoming homeless [3,4] and are at high risk for suicide [4-8]. The toxic effects of the everyday stress of homelessness are devastating. Reducing anxiety among homeless youth could have numerous health benefits given that anxiety and mood disorders are the leading causes of disability worldwide [9,10].

A growing body of evidence supports the benefits of meditation for reducing stress and improving mental and physical health, both immediately following meditation practice and over longer periods of follow up [11,12]. Mindfulness-based interventions (MBI) were developed to help individuals manage stress or illnesses, and are typically delivered in a group format that integrates meditation with mindfulness skills training [13,14]. Randomized controlled trials of MBI have demonstrated positive effects on reducing depression and anxiety symptoms [15,16]. Virtual reality (VR) meditation has recently become more widely available through commercialized apps that can be downloaded to smartphones or VR headsets and can provide users with an immersive guided meditation experience. The addition of VR meditation to MBI in adults with generalized anxiety disorder had higher retention than those who received MBI only [17]. VR meditation could promote greater engagement with MBI or other therapies, giving patients an opportunity to practice mindfulness skills in a setting removed from everyday distractions. However, we are unaware of any prior studies that have tested the feasibility of VR meditation for youth experiencing homelessness.

We assessed the feasibility of delivering one session of VR meditation and collecting momentary stress-related outcomes, including self-reported anxiety and physiologic measures of stress (salivary cortisol), among homeless youth. We focused the present study on momentary outcomes to assess the immediate changes following exposure to VR meditation, consistent with the design of other prior studies of meditation [12]. A convenience sample of 30 youth was recruited from a homeless youth drop-in center and enrolled in this randomized study of two meditation conditions (VR meditation and audio meditation as the meditation control) and VR-guided images as an attention control. The objectives of the study were to describe feasibility and changes in self-reported anxiety and salivary cortisol before and after intervention sessions along with interest in future use of VR by homeless youth. Assessing the feasibility of research procedures in marginalized populations, particularly among homeless youth, is critical given the potential for their greater mistrust and reluctance to accept interventions [18]. Our analysis compared pre and post differences in outcomes for the VR meditation group to the audio meditation group, and a secondary analysis was performed to compare the VR meditation group to the VR imagery group. We also describe qualitative findings regarding the experience of the youth with using the VR headset among those who received VR meditation or VR imagery.

## Methods

### Study Sample

A convenience sample of youth was recruited from a homeless youth drop-in center in a midwestern city in the United States. The drop-in center provides youth with access to food, laundry, and shower facilities, and recreational activities such as television, checking out books, and playing board games or video games. Drop-in center staff provide case management and link youth to community resources, including two full-time therapists who provide crisis counseling. Youth who attended the drop-in center were approached by research staff. Youth were eligible if they were between the ages of 18 and 24 years and met the current federal McKinney-Vento Act definition of homelessness in which they lacked a fixed, regular, and adequate nighttime residence [19]. Youth who were eligible and interested in the study consented to participate. The Institutional Review Board at The Ohio State University approved the study.

### Data Collection Procedures

Data collection occurred over 2 visits. At visit 1, subjects completed the baseline survey and were randomized. Participants who were randomized to one of the VR interventions were also provided a demonstration of the VR platform. Visit 2 occurred 1-3 days later and included the collection of a brief survey and 4 saliva samples before and after the intervention. The first saliva sample was collected immediately after the preintervention survey, sample 2 was collected 5 minutes later, sample 3 was collected immediately after the intervention, and sample 4 was collected 15 minutes later. Participants were compensated in person at the end of each data collection visit with cash in the amount of US $10 for visit 1 and US $40 for visit 2. As with all study measures, participants were free to decline any measure (including saliva collection).

### Intervention Conditions

Participants were randomly assigned at the first visit to one of three possible intervention groups: (1) VR meditation, (2) audio meditation as the meditation control, or (3) VR imagery as the attentional control [20]. The research study provided participants with one-time access to the Oculus Go headset in a private setting during the research appointment. For VR meditation, participants completed a guided meditation exercise with their choice of natural scenery (“Guided Meditation VR”) on the Oculus Go headset [21]. Guided Meditation VR was selected because it is a widely used platform, readily available for download, and has high-quality design features grounded in the core principles of meditation shown to improve outcomes related to stress [15,22]. Guided Meditation VR provides an immersive experience of guided meditation in which users complete an exercise in mindfulness meditation in natural scenes (eg, beach, mountains, forest) with a 360-degree field of view that allows participants to feel present in the scene as they complete the exercise. The audio meditation was accessed through a website and participants used headphones to listen to 10 minutes of a guided mindfulness meditation exercise [23]. The audio meditation was similar to the VR meditation with a focus on increasing relaxation and calmness through guided awareness of breath, body scans, and a nonjudgmental attitude toward distractions. However, the exact phrasing and voice instructions on meditation varied between the audio and VR meditations. Participants assigned to VR imagery used the Oculus Go headset to view historic images and text (Looking Glass VR) [20].

Instructions for how to navigate the platforms were given at the first visit. At the second visit, participants completed their randomly assigned intervention. There was some variation in the number of minutes that each intervention condition lasted. The meditation conditions lasted a set number of minutes, both approximately 10 minutes, whereas the VR imagery condition was self-directed. However, the timing given to access all modules was selected a priori so that all subjects had the same amount of time to complete or explore the modules (10-15 minutes).

### Measures

#### Feasibility Measures

Several additional measures were assessed to evaluate aspects of VR feasibility in the population and were collected from those who participated in either of the VR conditions (VR meditation or VR imagery) at the second visit, but not audio meditation. First, the feasibility of VR use was assessed by return participation rates and with open-ended questions assessing previous experience with VR, whether youth would be interested in using VR in the future, and how individuals felt their stress level changed following VR use (see Multimedia Appendix 1). In addition, three scaled items that are frequently used in VR research [24] were adopted to measure the sense of VR “presence” or “immersion” during use, including (1) sense of “being there,” (2) how close to reality the VR experience was felt to be, and (3) whether VR felt more like images that they saw or an experience they had. These items are scored on a scale from 1 to 7, with higher scores indicating a more immersive experience. These items were included to evaluate whether there were differences in the two VR groups in terms of how “real” the experience was for participants, aside from the differences in content.

#### Self-Reported Anxiety

Anxiety was assessed pre and post intervention at visit 2 with the 6-item short form of the State-Trait Anxiety Inventory-6 (STAI-6) [25], a validated and widely used instrument to measure momentary anxiety [25,26]. Each item on the STAI-6 asks subjects to rate how they feel (eg, “calm,” “relaxed,” “worried”) in that moment on a 4-point Likert scale ranging from “not at all” to “very much.” Following scoring instructions, positive feelings were reverse-scored, all items were summed, and the total was multiplied by 20 and divided by 6 to obtain the total score. The STAI-6 score ranges from 20 to 80 with higher scores indicating higher levels of anxiety.

#### Physiologic Stress

Physiologic stress was measured using salivary cortisol collected pre and post intervention at visit 2 via unstimulated passive saliva drool and stored at –20°C. Assays were conducted using Salimetrics Cortisol Enzyme Immunoassay Kits. The interassay coefficient of variation was 2.9% and the intraassay coefficient of variation was 8.9%. Similar to the STAI-6, salivary cortisol captures momentary stress given the physiologic response of cortisol to stress exposures and was used as a biomarker comparison to self-reported anxiety.

#### Baseline Measures

At visit 1, baseline measures were collected, including demographic characteristics (age, sex, and race), and self-reported lifetime diagnoses of mental health and substance use disorders, including depressive disorder, anxiety disorder, attention deficit and hyperactivity disorder, posttraumatic stress disorder, bipolar disorder, schizophrenia, personality disorder, alcohol use disorder, or drug use disorder. In addition, participants were asked to self-report any previous month use of alcohol, marijuana, illicit drugs, or tobacco (including cigarettes or vaping).

### Analysis

Descriptive statistics were used to describe self-reported demographic and clinical characteristics across groups, as well as average pre and post intervention values for anxiety and cortisol measures (log-transformed due to the skewed distribution). Feasibility of VR use was assessed based on summaries of participation rates and how positively youth reported the VR experience to be (both qualitative and scaled presence measures). Cohen *d* was calculated to estimate the effect size on anxiety and cortisol outcomes for VR meditation relative to other groups. Linear regression analyses were used to evaluate significant differences between intervention groups in pre and post intervention changes in anxiety scores, and separately for log-transformed salivary cortisol levels (comparing sample 2 vs 3, and sample 2 vs 4). Analyses compared differences in pre and post changes for the VR meditation group relative to the audio meditation group, and the VR meditation group relative to the VR imagery group. Because of varying preintervention anxiety scores and cortisol levels across groups, linear regression analyses included the preintervention value (anxiety score or cortisol level) as a covariate. Significant differences between groups were evaluated at *P*<.05 and all analyses were performed in SAS version 9.4.

## Results

Among the 35 youth who were screened for eligibility, 30 consented to participate and were randomized to study conditions (Multimedia Appendix 2 and Table 1). Over the two study visits, only 1 participant, who was assigned to the VR meditation group, was lost to follow up and did not return for study visit 2. Among the youth who completed either VR condition (n=18), only 1 participant who had a seizure disorder chose not to participate in their assigned condition (VR meditation) owing to concerns that VR could trigger a seizure. 

In open-ended questions, almost half of the youth had used VR in the past (n=8) and all reported interest in future use. The reported sense of “presence” was high in both groups, with a mean VR presence score of 5.3 (SD 2.2) for “how close it felt to reality,” 6.4 (SD 1.5) for whether the virtual environment felt more like “images that you have seen or a place that you have visited,” and 6.8 (SD 0.5) for “being there.” All youth in the VR meditation group reported that their stress had “dropped” or that they felt “calm.” However, only 2 participants in the VR images group reported feeling “relaxed” or “calm,” whereas most participants reported a positive experience such as being “entertained.”

**Table 1:** Sample characteristics for all participants and by intervention type.

Characteristics		Total sample (N=29)	VR^a^ meditation (n=8)	Audio meditation (n=11)	VR imagery (n=10)	*P* value^b^
Age (years), mean		21.6	21.9	21.7	21.2	.71
Male, n (%)		15 (52)	5 (63)	6 (55)	3 (30)	.72
**Race, n (%)**						.68
	White	6 (21)	2 (25)	3 (27)	1 (10)	
	Black	16 (55)	3 (38)	6 (55)	7 (70)	
	Mixed	7 (24)	3 (38)	2 (18)	2 (20)	
**Mental health diagnosis^c^, n (%)**						
	Any mental health disorder	22 (76)	6 (75)	7 (64)	9 (90)	.48
	Depressive disorder	17 (59)	6 (75)	6 (55)	5 (50)	.64
	Anxiety disorder	15 (52)	4 (50)	5 (46)	6 (60)	.89
	ADHD^d^	15 (52)	5 (63)	5 (46)	5 (50)	.81
	Posttraumatic stress disorder	13 (45)	4 (50)	4 (36)	5 (50)	.80
	Bipolar disorder	15 (52)	5 (63)	5 (46)	5 (50)	.81
	Schizophrenia	3 (10)	1 (13)	2 (18)	0 (0)	.47
	Personality disorder	6 (21)	2 (25)	2 (18)	2 (20)	>.99
**Substance use disorder diagnosis^e^, n (%)**						
	Illicit drug use disorder	4 (14)	1 (13)	2 (18)	1 (10)	>.99
	Alcohol use disorder	3 (10)	0 (0)	3 (27)	0 (0)	.09
	Any past-month alcohol use	14 (48)	6 (75)	5 (46)	3 (30)	.19
	Any past-month marijuana use	17 (59)	6 (75)	7 (64)	4 (40)	.36
	Any past-month illicit drug use (not including marijuana)	3 (10)	0 (0)	2 (18)	1 (10)	.76
	Any past-month tobacco use	25 (86)	7 (88)	9 (82)	9 (90)	>.99

^a^VR: virtual reality.

^b^*P* values for assessing group differences: small sample sizes limited the power to detect differences; one-way analysis of variance was conducted for age×randomization and the Fisher exact test was performed for all other comparisons by randomization.

^c^Mental health diagnoses are self-reported lifetime diagnosis of a mental health condition.

^d^ADHD: attention deficit/hyperactivity disorder.

^e^Substance use disorder diagnoses are lifetime; substance use frequency is based on a past-month timeframe.

Among the youth who completed their assigned intervention (n=29; VR or non-VR), mean anxiety scores declined in all groups, with the greatest difference observed in the VR meditation group (Table 2). However, the pre and postintervention difference in log-transformed salivary cortisol values had little variation across groups. The effect sizes for the log-transformed cortisol difference for the VR meditation group relative to the audio meditation group were small with Cohen *d* of 0.08 (sample 2 vs 3) and 0.43 (sample 2 vs 4). 

**Table 2:** Mean (SD) scores for anxiety and log-transformed cortisol levels pre and post intervention by intervention group.

Variable	VR^a^ meditation (n=8)	Audio meditation (n=11)	VR imagery (n=10)
Anxiety score pretest^b^	43.8 (14.9)	38.8 (17.5)	29.7 (8.4)
Anxiety score posttest^c^	32.9 (9.8)	33.0 (12.7)	24.7 (5.9)
Anxiety score difference	10.8 (8.3)	5.8 (8.9)	5.0 (8.8)
Log cortisol sample 1 pretest^d^	–1.52 (0.56)	–1.31 (0.56)	–1.40 (0.46)
Log cortisol sample 2 pretest^d^	–1.35 (0.64)	–1.15 (0.61)	–1.43 (0.50)
Log cortisol sample 3 posttest^e^	–1.48 (0.86)	–1.25 (0.59)	–1.31 (0.50)
Log cortisol sample 4 posttest^e^	–1.36 (0.82)	–1.36 (0.72)	–1.47 (0.34)
Log cortisol sample 2 vs 3 difference score	0.127 (0.36)	0.095 (0.42)	–0.121 (0.36)
Log cortisol sample 2 vs 4 difference	0.004 (0.39)	0.205 (0.54)	0.045 (0.41)

^a^VR: virtual reality.

^b^Collected before the intervention.

^c^Collected after the intervention at visit 2.

^d^Salivary samples collected before the intervention at visit 2.

^e^Salivary samples collected after the intervention at visit 2.

Based on linear regression analyses, there were no significant differences between the VR meditation group and other groups in pre and post differences for any anxiety or cortisol outcomes (Table 3). Relative to other groups, the VR meditation had a modest effect size relative to audio meditation and VR imagery on anxiety scores, but comparisons between groups in salivary cortisol depending on the timing of the saliva sample were less consistent (Table 4). Audio meditation relative to VR imagery had a very small effect size for anxiety and a modest effect size for salivary cortisol.

**Table 3:** Linear regression analyses examining anxiety and log-transformed salivary cortisol differences pre and post intervention.

Comparison	Model 1^a^			Model 2^b^			Model 3^b^		
	β	SE	*P* value^c^	β	SE	*P* value	β	SE	*P* value
VR^d^ meditation (n=8) vs audio meditation (n=11, reference group)	3.13	2.82	.28	.04	0.19	.84	–.19	0.23	.43
VR meditation (n=8) vs VR imagery (n=10, reference group)	–1.80	3.34	.59	.04	0.04	.30	–.06	0.07	.38

^a^Model 1: difference in pre and post State-Trait Anxiety Inventory-6 anxiety scores reflect measures collected before vs after the interventions at visit 2, controlling for preintervention anxiety levels.

^b^Models 2/3: difference in pre and post log cortisol values reflect measures collected before (sample 2) vs after (sample 3/sample 4) the interventions at visit 2, controlling for preintervention logged cortisol levels from sample 2.

^c^*P* values are based on the Wald test, comparing VR meditation to the referent group (audio meditation or VR imagery).

^d^VR: virtual reality.

**Table 4:** Effect sizes (Cohen d) in comparisons between intervention groups.

Comparison	Anxiety pretest vs posttest	Log cortisol sample 2 vs sample 3	Log cortisol sample 2 vs sample 4
VR^a^ meditation vs audio meditation	0.58	0.08	0.43
VR meditation vs VR imagery	0.68	0.69	0.10
Audio meditation vs VR imagery	0.09	0.54	0.33

^a^VR: virtual reality.

## Discussion

Among youth experiencing homelessness, we evaluated two different meditation platforms (VR meditation and audio meditation) and a VR platform with imagery. Overall, the interventions appeared to be feasible to deliver, and the youth rated their experiences positively. Importantly, the study had a high degree of retention in a high-risk and difficult-to-engage population, with all but one youth completing their assigned VR condition and all youth completing comparison group interventions. We observed a moderate positive effect on anxiety for VR meditation relative to audio meditation; although the difference in anxiety changes was not significant in our small sample, the results suggest that a moderate effect size may be possible in a larger trial. These results thus demonstrate the potential of using VR meditation in a study of the homeless youth population. In addition to the potential benefits for reduced anxiety, the youth also reported interest in using VR in the future. Collection of physiologic measures of stress also appeared feasible, with all youth providing samples to assess salivary cortisol. Together, these results suggest that the VR meditation and comparison interventions are feasible to complete and the measures to capture momentary changes in stress-related outcomes are also feasible to collect in a high-risk population.

Although self-reported anxiety decreased in all groups, salivary cortisol levels did not decline, and there were no statistically significant differences in anxiety or cortisol levels between the VR meditation group and the referent audio meditation and VR imagery groups. The small sample of youth limited our ability to detect significant differences, and it is possible that youth may need longer, repeated sessions or mindfulness skills training [27] for a significant beneficial change to be observed. In addition, the demographic characteristics of the intervention groups were not balanced by sex and race/ethnicity.

Nevertheless, the present study is the first to demonstrate the feasibility of recruiting youth experiencing homelessness to participate in a study evaluating different VR platforms and modes of meditation (VR vs audio). The study had a high retention rate, and the youth were enthusiastic about using VR platforms in the future if made available to them. However, there was one participant who declined participation in VR meditation due to a self-reported medical condition that precluded their participation (epilepsy), and studies that use this technology in the future may need to consider excluding participants with this or other conditions that limit VR participation. Although the high retention rate could reflect the increased monetary incentive amount at visit 2 compared to visit 1, this is a strategy often used to reduce attrition in difficult-to-engage populations of homeless youth [28,29]. Moreover, the increased incentive amount was used to compensate for the greater burden of data collection and was the same for all groups. There was a trend toward a greater reduction in anxiety among the VR meditation group relative to the audio group. These results suggest that further study is warranted in a larger sample and over a longer follow-up period.

## Appendix

Multimedia Appendix 1 Open-ended questions about virtual reality (VR) experience.

Multimedia Appendix 2 Consolidated Standards of Reporting Trials (CONSORT) flow diagram of the study.

## Abbreviations

MBI: mindfulness-based intervention
STAI-6: State-Trait Anxiety Inventory-6
VR: virtual reality
