# Supplementary material for: Virtual Reality Meditation Among Youth Experiencing Homelessness: Pilot Randomized Controlled Trial of Feasibility
Source: JMIR Ment Health. 2020 Sep 24;7(9):e18244. doi: 10.2196/18244 (PMC7545327; doi:10.2196/18244)
Supplement: Multimedia Appendix 1 [file mental_v7i9e18244_app1.docx]

Open-ended questions for VR Experience:

1. Tell me about other times you have used virtual reality. What past experience have you had with Virtual Reality?
2. How did you feel before using the Virtual Reality tool?
3. How did you feel while using the Virtual Reality tool?
4. How did you feel after using the Virtual Reality tool?
5. How did your level of stress change after using the Virtual Reality tool?
6. Would you use Virtual Reality tools, such as those you tried today, on a regular basis if it was available to you?
