# Supplementary material for: Virtual Reality Meditation Among Youth Experiencing Homelessness: Pilot Randomized Controlled Trial of Feasibility
Source: JMIR Ment Health. 2020 Sep 24;7(9):e18244. doi: 10.2196/18244 (PMC7545327; doi:10.2196/18244)

CONSORT
TRANSPARENT REPORTING of TRIALS

CONSORT 2010 Diagram

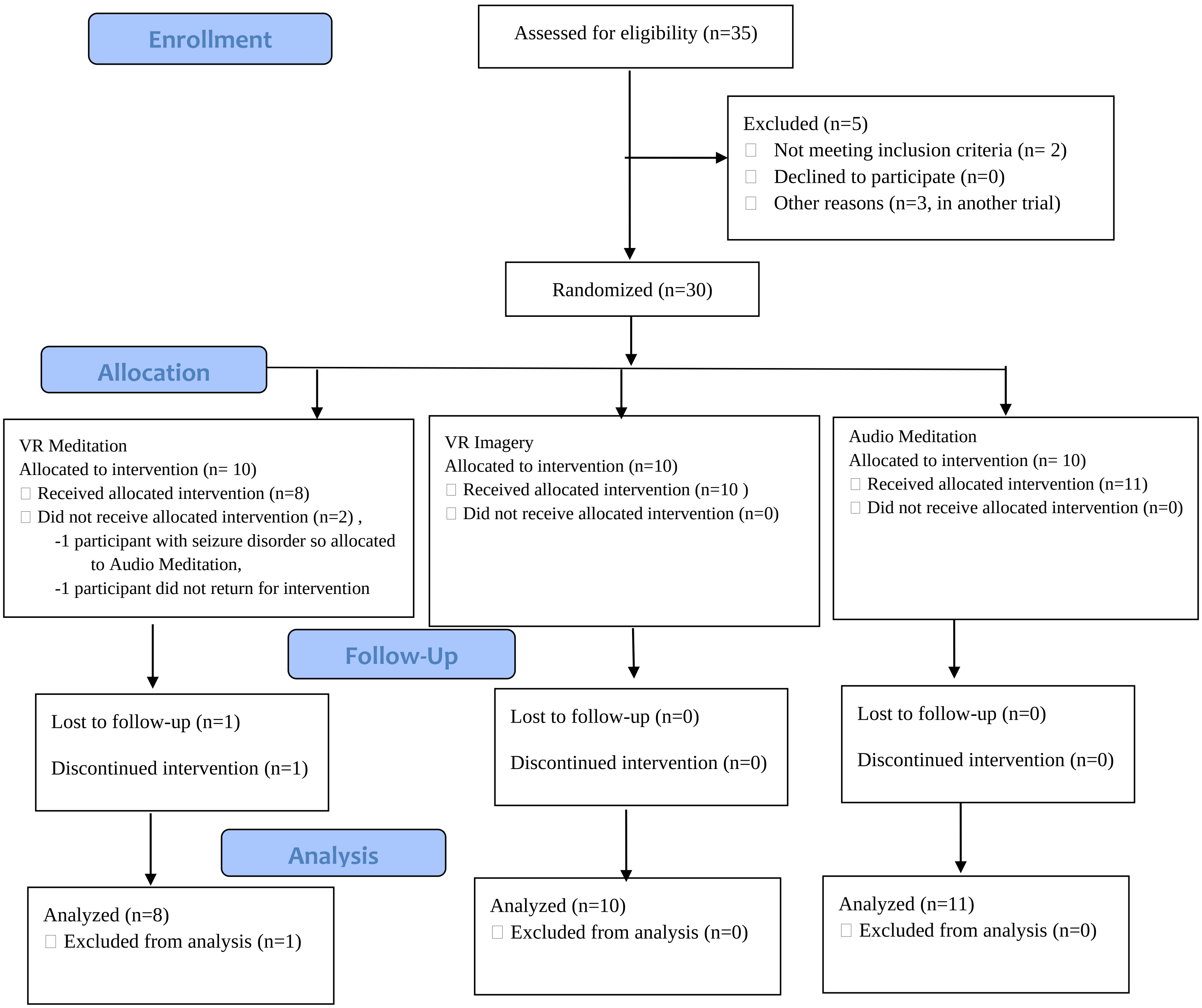

Supplement: Multimedia Appendix 2 [file mental_v7i9e18244_app2.pdf]
